# Confusion as an Unusual Presentation of Infective Endocarditis

**DOI:** 10.7759/cureus.20809

**Published:** 2021-12-29

**Authors:** Mohammed Dabbous, Michel Saba, Said El Orra, Claudette Najjar

**Affiliations:** 1 Cardiology, Lebanese University Faculty of Medicine, Beirut, LBN; 2 Internal Medicine, Beirut Arab University, Beirut, LBN; 3 Cardiology, Beirut Cardiac Institute, Beirut, LBN

**Keywords:** case report, vegetation, embolic event, confusion, infective endocarditis

## Abstract

Infective endocarditis (IE) is a rare infection of the inner lining of the heart and valves, mainly affecting those with pre-existing heart problems. Patients usually present with fever and other non-specific systemic symptoms such as malaise, myalgia, and night sweats. However, IE may have unusual presentations, making its diagnosis even more challenging. Here, we report an unusual case of IE presenting as confusion.

A 51-year-old man presented to the emergency department complaining of confusion for three days. Upon physical examination, there was an evident holosystolic murmur at the apex radiating to the axilla and an early decrescendo diastolic murmur at the left lower sternal border. Laboratory tests including white blood cell count and C-reactive protein were elevated. Transthoracic and transesophageal echocardiogram showed severe mitral regurgitation and aortic regurgitation, in addition to the presence of a mobile mass suspected to be vegetation on each of the mitral and aortic valves. Magnetic resonance imaging of the brain was performed which revealed ischemic lesions of possible embolic origin. Mitral and aortic valve replacement was performed successfully, and the patient recovered well.

Our case emphasizes the possibility of unusual presentations in patients with IE, with confusion being one of them. It is important for physicians to always consider the diagnosis of IE in patients presenting with neurological symptoms of unclear origin.

## Introduction

Infective endocarditis (IE) is a rare disease with an incidence of around three to seven per 100,000 person-years [1]. There are multiple risk factors for IE including congenital heart disease, rheumatic valve disease, complications following mechanical valve replacement or cardiac device implantation (such as infection and formation of hematoma), mitral valve prolapse, immunosuppression, and intravenous drug use [2]. Patients with IE can have non-specific presentations such as fever, chills, weight loss, and loss of appetite [3]. Several cases of IE have been previously reported describing unusual presentations, including polyarthritis, dysphagia, meningitis, leg weakness, and atrioventricular (AV) block [4-8]. Here, we report a case of confusion as an unusual presentation of IE.

## Case presentation

A 51-year-old male, who was previously healthy, smoker, and moderate alcohol consumer, was admitted to the emergency department due to agitation and confusion for three days. The patient denied any agitation or confusion but admitted that he “wasn’t feeling well” and decided to take ampicillin. The delayed presentation to the hospital was explained by the fear of the patient that he might get infected by the coronavirus disease 2019 (COVID-19). According to his family, the confusion and agitation lasted for a few hours, recurred throughout the day, and were not associated with headache, syncope, disorientation, or amnesia. The patient denied a history of intravenous drug use, dental procedures, or suffering from a head trauma prior to presentation.

On physical examination, the patient was alert and oriented to time and place, with a normal Glasgow Coma Scale score of 15. His vital signs were normal, except for a fever of 38.5°C. Cardiac examination revealed an audible grade 3 holosystolic murmur at the apex radiating to the axilla and a grade 3 early decrescendo diastolic murmur at the left lower sternal border. The rest of the examination was unremarkable.

**Figure 1:**
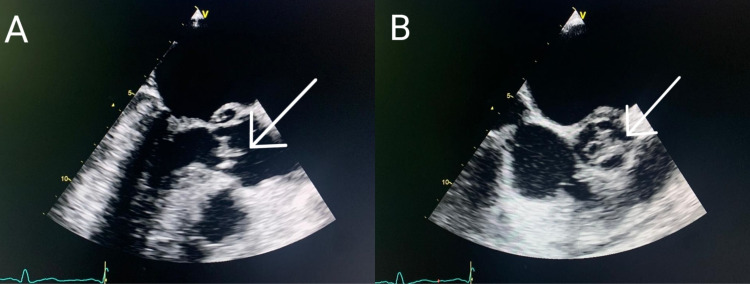
Transesophageal echocardiogram. A: vegetation at the mitral valve. B: vegetation at the aortic valve.

Laboratory tests showed elevated white blood cell (WBC) count (12,000/mm^3^) and C-reactive protein (CRP) (15 mg/L). Erythrocyte sedimentation rate (ESR), random blood glucose test, renal function tests, liver function tests, procalcitonin, lactate, and arterial blood gases were normal. As a part of the workup, a carotid artery Doppler ultrasound was done which showed no abnormality. A transthoracic echocardiogram (TTE) was then performed which showed severe aortic and mitral regurgitation. At this point, IE was suspected. Hence, blood cultures were obtained and a transesophageal echocardiogram (TEE) was performed which confirmed the findings of the TTE, in addition to the presence of thickened anterior leaflet of the mitral valve, a draining abscess at the aortic valve, and a mobile mass suspected of vegetation at the level of the mitral and aortic valves (Figures 1A, 1B).

**Figure 2:**
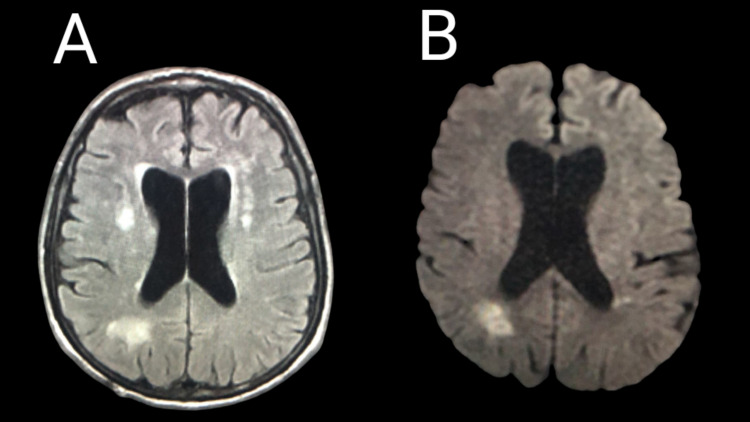
MRI of the brain at the level of the lateral ventricles. A: axial T2-FLAIR imaging showing multiple bilateral and peri-ventricular cortico-subcortical foci of high-intensity signal. B: axial DWI showing restriction of diffusion. DWI: diffusion-weighted imaging; MRI: magnetic resonance imaging; T2-FLAIR: T2-weighted fluid-attenuated inversion recovery

Magnetic resonance imaging (MRI) of the brain at the level of the lateral ventricles revealed multiple bilateral and peri-ventricular cortico-subcortical foci of high-intensity signal on T2-weighted fluid-attenuated inversion recovery (T2-FLAIR) imaging, as well as restriction of diffusion on diffusion-weighted imaging (DWI), denoting the presence of acute infarcts possibly due to embolic events (Figures 2A, 2B). Blood cultures were negative.

The diagnosis of IE was made and empirical vancomycin was initiated. Five days after the initial presentation, the patient’s condition deteriorated. He developed AV block, cardiac failure, and eventually cardiogenic shock, for which he was transferred to the intensive care unit (ICU). COVID-19 infection was suspected at this stage, but repeated polymerase chain reaction (PCR) tests for COVID-19 were negative. However, we believed that the results were false-negative as there was an increasing number of infected patients in the hospital at the time. After stabilizing the patient, he was scheduled for mitral and aortic replacement surgery, during which surgical cultures were taken from both valves. The surgery was successful. However, five days post-surgery, the patient developed pneumonia and pleural effusion which were treated with a six-week course of intravenous levofloxacin.

Surgical cultures returned negative. After seven weeks post-surgery, the patient was discharged home. Echocardiographic follow-up every three months for a year showed adequately functioning prosthetic valves.

## Discussion

Despite the advancements in the medical field over the years, the incidence of IE has not decreased, which may be attributed to more cases of IE being diagnosed, the increased number of intravenous drug users and implanted devices, the increase in infections caused by nosocomial organisms, and the higher life expectancy of patients [9].

*Staphylococcus *remains the main causative agent for IE. In the United States, studies have shown an increase in endocarditis caused by *Enterococcus*, coagulase-negative *Staphylococcus*, and *Staphylococcus aureus*. On the other hand, endocarditis caused by *Streptococcus viridans* and culture-negative endocarditis decreased. This can be explained by the increased number of intravenous drug users, increased vascular access device implantations, higher healthcare contact, and improved culture and laboratory tests [10].

IE arises following an injury to the valvular endothelium, making it a perfect medium for bacterial growth. Endothelial injury may be influenced by the presence of prosthetic valves, intravenous drug use, turbulent blood flow in a congenital heart defect, and inflammatory diseases such as rheumatic carditis [11].

Patients with IE commonly present with fever, chills, arthralgias, fatigue, and night sweats. Cardiac manifestations are usually seen as heart murmurs upon examination, as well as the presence of cardiac pathologies on echocardiogram. Patients may also present with signs of embolic events, manifesting as renal failure, cerebrovascular accident, and peripheral artery disease [9]. Patients with suspected IE should be asked about any history of heart diseases, history of dental procedure, presence of an indwelling catheter, and intravenous drug use [12]. Although neurological manifestation is a common finding in patients with IE, confusion is a rare presentation of IE. As seen in our patient, confusion can be caused by embolic events, as seen on the MRI findings.

The Modified Duke’s Criteria remains the method of choice for the diagnosis of IE [13]. Laboratory tests including WBC count, CRP, ESR, procalcitonin, bilirubin, and lactic acid may be elevated. However, this does not have much benefit in the diagnosis of IE [3]. Blood culture, one of the major criteria for the diagnosis of IE, is indicated in patients with fever of unknown source for 48 hours, murmur on auscultation, and valvular impairment evident on echocardiogram [2]. The results of the culture may not always be positive. This occurs either when the causative organism is an intracellular organism (such as *Bartonella *and *Coxiella* *burnetii*) or a fastidious organism (fungi and *HACEK *organisms) or when the patient had received antibiotics prior to blood collection [11].

In our case, the negative blood culture may be explained by the possible presence of a fastidious organism. However, the lack of hospital resources, particularly a special culture medium, made it difficult for us to identify the causative organism. It may also be explained by the antibiotics taken by the patient prior to his presentation. Furthermore, although the inflammatory markers wereslightly raised in our case, this did not influence our suspicion of IE. IE with low inflammatory markers is rare and can be encountered in immunocompromised patients and cases of IE caused by organisms such as *Coxiella burnetii* and *Bartonella henselae* [14]. Because our patient did not have immunodeficiency, the laboratory tests may be explained by the fastidious organism.

TTE remains the imaging of choice for the diagnosis of IE. TEE is usually performed following TTE for better evaluation. Although new imaging modalities have emerged (e.g., positron emission tomography-computed tomography, MRI), there is a lack of evidence for favoring them over the traditional echocardiogram [2].

Embolic events appear to be a major problem in IE. In a retrospective study, 499 out of 1,456 cases of IE were associated with at least one embolic event. In the same study, factors associated with embolic events included vegetation size (>13 mm), the presence of prosthetic valve, right-sided IE, and the presence of *Staphylococcus aureus* as a causative agent [15].

Regarding the treatment of IE, antibiotics are introduced after blood cultures are obtained. The type of antibiotic used depends on the result of the culture. However, if the patient is very ill, empirical antibiotic therapy may be initiated [12]. Early surgical intervention is recommended in cases of IE associated with recurrent embolic events, infections caused by a resistant organism, abscess, heart failure, or AV block [13]. In our case, the development of heart failure and AV block five days after the presentation were clear indications for early surgical intervention.

Early surgery may have a better prognosis in the management of IE. This was supported by the Early Surgery Versus Conventional Treatment in Infective Endocarditis (EASE) trial. Within the first five years since the diagnosis of IE, patients in the conventional therapy group had a higher rate of recurrence of IE, embolic events, and endpoint of deaths compared to the early surgery group [16].

Several factors are thought to be associated with ICU admission in patients with IE. According to a retrospective study conducted among 228 patients with IE, the notable factors included neurological complications (occurring in 40% of the patients), congestive heart failure (28%), and septic shock (26%) [17].

There is a debate on whether antibiotic prophylaxis against *Streptococcus viridans *is effective. The American Heart Association (AHA) has emphasized that there is not enough evidence proving its effectiveness. However, it may be considered solely in patients belonging to the following high-risk groups: congestive heart failure, cardiac transplantation developing into valvular dysfunction, relapsing or recurrent IE, and prosthetic valves or cardiac devices. Dental hygiene is considered to be crucial. The AHA recommends a dental examination twice a year for high-risk patients [18].

## Conclusions

Physicians need to consider the possibility of unusual presentations in patients with IE. As described in our case, the patient’s first presentation was confusion, which was eventually diagnosed as IE. Specifically, physicians are encouraged to suspect IE in patients presenting with neurological manifestations of an unknown cause.
